# Facilitating question identification through natural intensity eyebrow movements in virtual avatars

**DOI:** 10.1038/s41598-023-48586-4

**Published:** 2023-12-02

**Authors:** Naomi Nota, James P. Trujillo, Vere Jacobs, Judith Holler

**Affiliations:** 1 Donders Institute for Brain, Cognition, and Behaviour, Nijmegen, The Netherlands; 2 Max Planck Institute for Psycholinguistics, Nijmegen, The Netherlands; 3 Faculty of Arts, Radboud University, Nijmegen, The Netherlands

**Keywords:** Human behaviour, Language

## Abstract

In conversation, recognizing social actions (similar to ‘speech acts’) early is important to quickly understand the speaker’s intended message and to provide a fast response. Fast turns are typical for fundamental social actions like questions, since a long gap can indicate a dispreferred response. In multimodal face-to-face interaction, visual signals may contribute to this fast dynamic. The face is an important source of visual signalling, and previous research found that prevalent facial signals such as eyebrow movements facilitate the rapid recognition of questions. We aimed to investigate whether early eyebrow movements with natural movement intensities facilitate question identification, and whether specific intensities are more helpful in detecting questions. Participants were instructed to view videos of avatars where the presence of eyebrow movements (eyebrow frown or raise vs. no eyebrow movement) was manipulated, and to indicate whether the utterance in the video was a question or statement. Results showed higher accuracies for questions with eyebrow frowns, and faster response times for questions with eyebrow frowns and eyebrow raises. No additional effect was observed for the specific movement intensity. This suggests that eyebrow movements that are representative of naturalistic multimodal behaviour facilitate question recognition.

## Introduction

Recognizing social actions^1^ (similar to ‘speech acts’^2,3^) is a crucial part of having a successful conversation, as they indicate the goal of the speaker’s utterance. For instance, the utterance “They changed their hair colour” could either be a question or a statement depending on the speaker’s intended message with their utterance in the conversational context. Conversation consists of fast-turns between speakers, and swiftly identifying the speaker’s intended message may help to provide a fast response^1,4–7^. This fast timing is typical for fundamental social actions like questions, since waiting too long before answering can indicate a dispreferred response^8^ (e.g., declining an invitation).

In multimodal face-to-face interaction^7,9^, the face is an important source of visual signalling, and specific facial signals were found to associate with particular social actions^10–13^. Crucially, prevalent facial signals in conversation such as eyebrow movements were often associated with questions across various spoken and signed languages^10,12–22^, and were shown to occur early^12,13,23^. This study aims to investigate early eyebrow movements in the rapid identification of questions.

Past experimental research testing language comprehension frequently found that eyebrow movements like eyebrow frowns and raises made questions recognizable when they accompanied the spoken utterance^14,24–28^. A recent study^24^ investigated the contribution of eyebrow frowns and raises in question identification, taking detailed features of natural conversation into account, such as different question types, word lengths, as well as different utterance and facial signal durations. Participants watched videos of avatars (virtual reality characters), whose visual and spoken behaviour was derived from spontaneously produced conversations from a large face-to-face Dutch corpus. Results showed higher accuracies and faster response times for questions with eyebrow frowns, but not eyebrow raises, compared to questions without eyebrow movements, demonstrating the facilitative role of eyebrow movements for question identification.

While Nota et al.^24^ used stimuli that closely reflected naturalistic multimodal behaviour in terms of presence and timing of facial signals, the movement intensities were fixed on a maximum intensity setting, which might have enhanced certain facial signals when their movement intensities were originally lower in the corpus. If this is the case, then the facilitation effect observed in Nota et al.^24^ may be an artefact that disappears when introducing the variability of movement intensity inherent to natural facial expression. Alternatively, it may be that if natural variation is introduced, only specific eyebrow movement intensities mark questionhood, which would provide a more refined understanding of how facial signals contribute to face-to-face interaction. Therefore, an open question is whether eyebrow movements facilitate question identification when they have natural movement intensities, and whether specific intensities are more helpful in detecting questions faster and more accurately.

### Current study

In the present study, we extend previous research on the effect of early eyebrow movements (or the dynamic shift in movement to create an eyebrow frown or eyebrow raise, which includes movement of the skin between or above the eyebrows as such) in the rapid identification of questions by using natural movement intensities. We addressed the following research questions:

1. Do eyebrow movements with natural movement intensities facilitate accuracy and speed of question identification?
2. Do specific eyebrow movement intensities result in higher accuracies and faster speed of question identification compared to other eyebrow movement intensities?

One possibility is that question-accompanying eyebrow movements with natural movement intensities will result in a stronger facilitation effect. The previous facilitation effect of eyebrow frowns in Nota et al.^24^ was observed while enhancing eyebrow movements to a fixed and maximum movement intensity. Therefore, another possibility could be that the natural movement intensities result in less facilitation, since they are less pronounced. However, the interplay between facial signals and questions may be more complex, since natural movement intensities introduce more variability, potentially making certain movement intensities stand out more in contrast to others.

Like Nota et al.^24^, to test and control for potential co-variables, we took into account differences in eyebrow movement onsets and verbal utterance onsets, eyebrow movement duration, question type, as well as utterance duration, and expected these variables to have similar effects. In addition, we collected different psycho-social questionnaires to provide a more comprehensive characterization of the sample.

Results will provide a conceptual advance by determining whether the exact intensity of a signal contributes to question identification. Crucially, this work provides important methodological insights for developing virtual characters, such as avatars or social robots, by showing whether the implementation of variable movement intensities is required to serve their natural pragmatic functions. In the same line, this work will inform the importance of tracking movement intensities when performing studies with motion tracking, or whether the presence and timing is sufficient to capture the signal. This study represents a significant departure from conventional approaches employed in previous studies, which typically relied on confederates or actors. Instead, the present study employed virtual avatars with speech and visual features that more closely reflected real-life multimodal behaviour, while simultaneously preserving the necessary level of experimental control for reliable data collection^29,30^.

## Results

### Accuracy

#### Effect of eyebrow movements

We first assessed whether the presence of eyebrow movements (frowns or raises) led to higher accuracies in categorizing an utterance as a question, as opposed to a statement. There was a significant main effect of eyebrow movement (*χ*^2^(2) = 11.71, *p* = 0.003). We then looked at whether specific differences between eyebrow movement onsets and verbal utterance onsets, or particular eyebrow movement durations, led to higher accuracies. There was no significant main effect of difference in onsets, nor of eyebrow movement duration.

The post-hoc analysis on the effect of eyebrow movements on accuracies revealed a significant difference between eyebrow frowns and no eyebrow movement (*β* = − 0.44, *SE* = 0.13, *z* = − 3.40*, p* = 0.002). However, there was no significant difference between eyebrow raises and no eyebrow movement.

**Figure 1:**
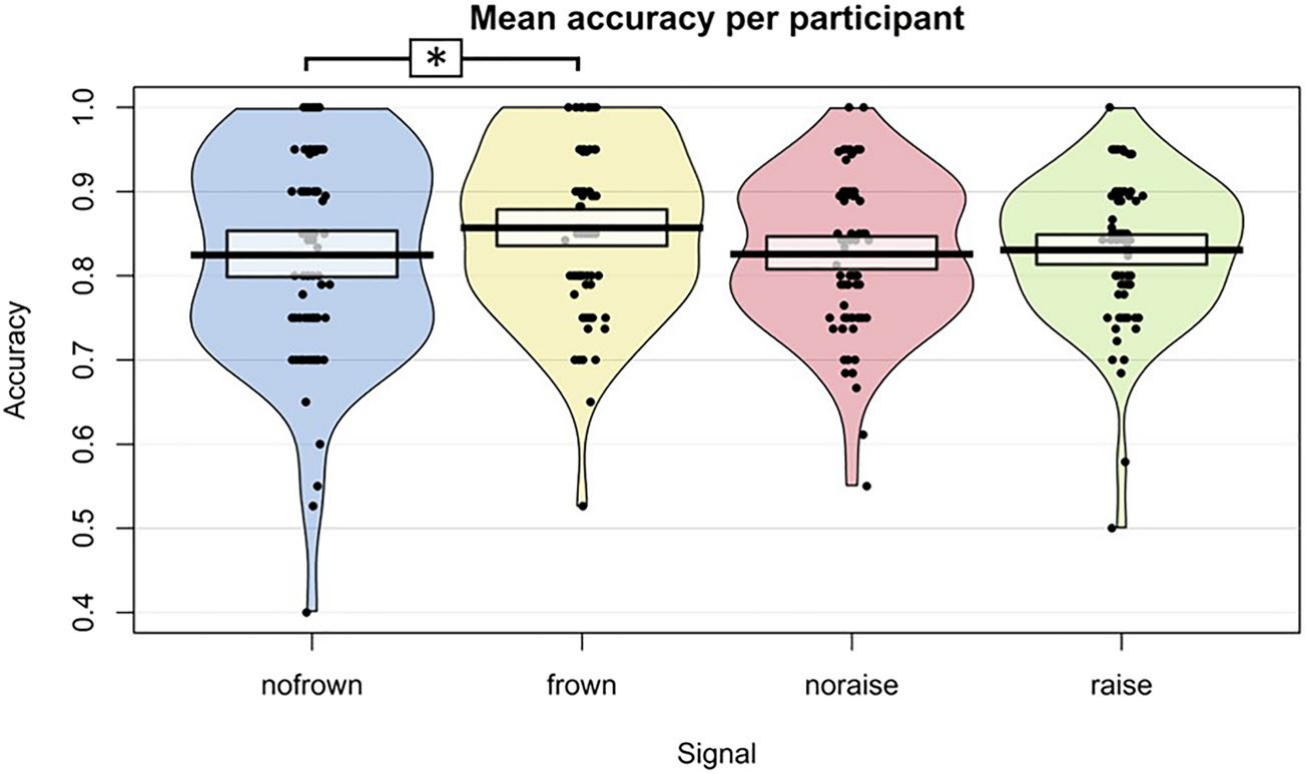
Overview of the accuracy results. For better visualization, absence of eyebrow movement is split in absence of eyebrow frown and absence of eyebrow raise. Pirate plots depict the distribution of the accuracies for absence of eyebrow frown movement, eyebrow frown movement, absence of eyebrow raise movement, and eyebrow raise movement. Individual dots represent overall mean accuracy for individual participants (raw data). Bars indicate means, beans (the oval shapes around the dots) indicate smoothed density, and bands (dark-coloured lines at the top of the bars) indicate the 95% Bayesian Highest Density Interval (HDI). Asterisks indicate a significant difference between conditions. The model equation was: Accuracy ~ Eyebrow movement (eyebrow frown, eyebrow raise, no eyebrow movement) + Difference onsets + Eyebrow movement duration + Question type (declarative, interrogative, non-clausal, tag-, wh-) + Utterance duration + (1 | Participant) + (1 | Item). Note that excluding the participant showing a mean accuracy below chance level in the ‘no frown’ condition in additional analyses did not change the significance of the effect of eyebrow movement, nor the significant difference between eyebrow frowns and no eyebrow movement (see the Open Science Framework project website for these additional exploratory analyses).

These results show that only the presence of eyebrow frowns resulted in higher accuracies in question identification compared to the absence of eyebrow movement, but not the presence of eyebrow raises (Fig. 1).

#### Effect of specific eyebrow movement intensities

When computing whether specific eyebrow movement intensities led to higher accuracies in categorizing an utterance as a question, as opposed to a statement, we observed no significant main effect of specific eyebrow movement intensity. When looking at Bayes factor, there was only anecdotal evidence in favour of a null effect of eyebrow movement intensity (BF = 1.29), indicating we do not have sufficiently conclusive evidence for supporting a null effect of specific movement intensity on accuracies.

#### Effect of question type and utterance duration

We tested whether specific question types led to higher accuracies, and found a significant effect of question type (*χ*^2^ (4) = 57.80, *p* < 0.001). Furthermore, we investigated whether specific utterance durations led to higher accuracies. We found a significant effect of utterance duration (*χ*^2^ (1) = 6.78, *p* = 0.009), showing that longer utterances resulted in higher accuracies.

The post-hoc analysis on the effect of question types on accuracies showed a significant difference between declarative and interrogative questions (*β* = − 3.93, *SE* = 0.54, *z* = − 7.22*, p* < 0.001), between declarative and wh-questions (*β* = − 3.62, *SE* = 0.49, *z* = − 7.42*, p* < 0.001), between interrogative and tag-questions (*β* = 2.77, *SE* = 0.53, *z* = 5.20*, p* < 0.001), and between tag- and wh-questions (*β* = − 2.46, *SE* = 0.48, *z* = − 5.15*, p* < 0.001).

### Response time

#### Effect of eyebrow movements

We first assessed whether the presence of eyebrow movements (frowns or raises) led to faster response times (RTs) when categorizing an utterance as a question, as opposed to a statement. There was a significant main effect of eyebrow movement (*χ*^2^ (2) = 22.40, *p* < 0.001). We then looked at whether specific differences between eyebrow movement onsets and verbal utterance onsets led to faster RTs. There was a significant main effect of difference in onsets (*χ*^2^ (1) = 72.90, *p* < 0.001), showing that larger differences in onsets resulted in faster RTs. When looking at whether particular eyebrow movements durations resulted in faster RTs, we observed a significant main effect of eyebrow movement duration (*χ*^2^ (1) = 3.87, *p* = 0.049). This shows that longer eyebrow movement durations resulted in faster RTs.

The post-hoc analysis on the effect of eyebrow movements on RTs revealed a significant difference between eyebrow frowns and no eyebrow movement (*β* = 0.04, *SE* = 0.01, *z* = 3.43*, p* = 0.002), and between eyebrow raises and no eyebrow movement (*β* = 0.04, *SE* = 0.01, *z* = 3.04*, p* = 0.007).

**Figure 2:**
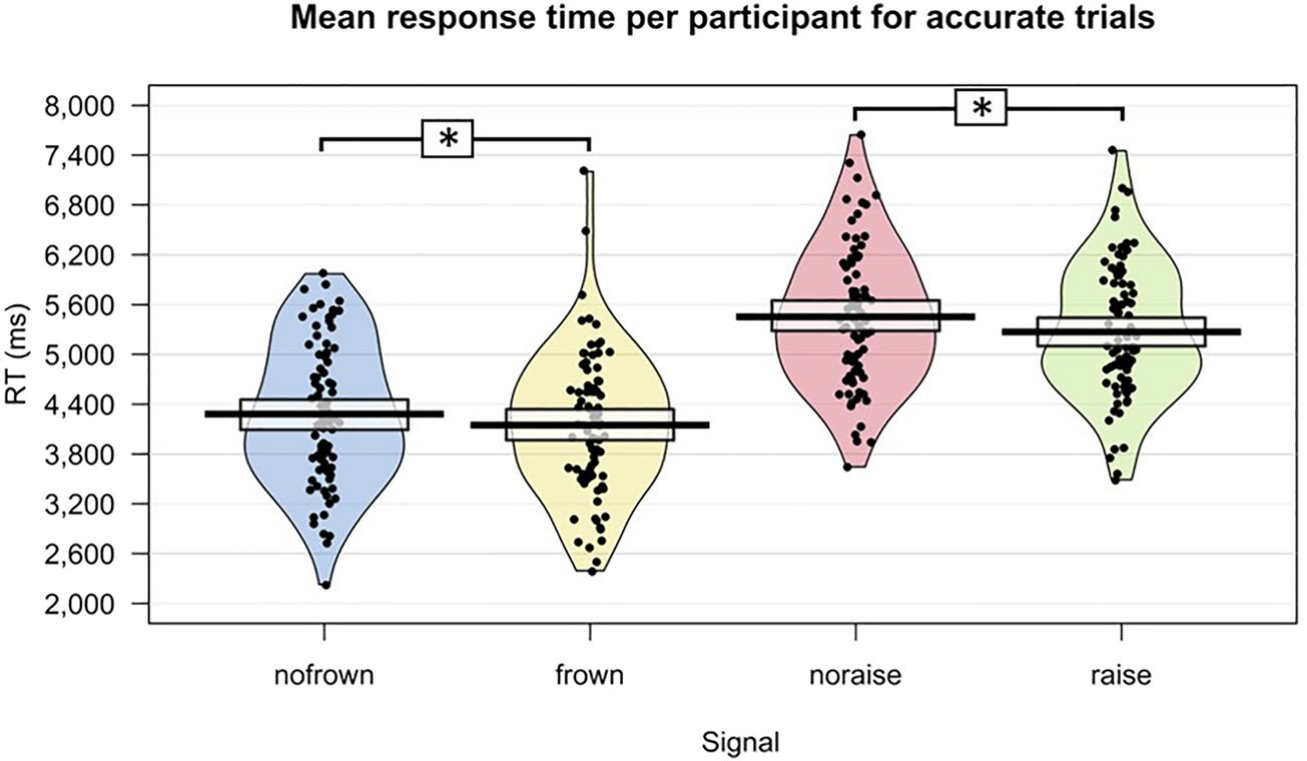
Overview of the RT results. For better visualization, absence of eyebrow movement is split in absence of eyebrow frown and absence of eyebrow raise. Pirate plots depict the distribution of the accuracies for absence of eyebrow frown movement, eyebrow frown movement, absence of eyebrow raise movement, and eyebrow raise movement. Individual dots represent overall mean RT for individual participants (raw data). Bars indicate means, beans (the oval shapes around the dots) indicate smoothed density, and bands (dark-coloured lines at the top of the bars) indicate the 95% Bayesian Highest Density Interval (HDI). Asterisks indicate a significant difference between conditions. The model equation was: RT ~ Eyebrow movement (eyebrow frown, eyebrow raise, no eyebrow movement) + Difference onsets + Eyebrow movement duration + Question type (declarative, interrogative, non-clausal, tag-, wh-) + Utterance duration + (1 | Participant) + (1 | Item). RT was measured from the beginning of the video until the button-press.

These results show that the presence of eyebrow frowns and the presence of eyebrow raises in questions resulted in faster RTs compared to the absence of eyebrow movement (Fig. 2).

#### Effect of specific eyebrow movement intensities

When computing whether specific eyebrow movement intensities led to faster RTs when categorizing an utterance as a question, as opposed to a statement, we observed no significant main effect of specific eyebrow movement intensity. When looking at Bayes factor, there was strong evidence in favour of a null effect of specific eyebrow movement intensity on RTs (BF = 28.59).

#### Effect of question type and utterance duration

We tested whether specific question types led to faster RTs, and found a significant effect of question type (*χ*^2^ (4) = 14.92, *p* = 0.005). Furthermore, we investigated whether specific utterance durations led to faster RTs. We found a significant effect of utterance duration (*χ*^2^ (1) = 27.55, *p* < 0.001), showing that longer utterances resulted in longer RTs.

The post-hoc analysis on the effect of question types on RTs showed a significant difference between declarative and interrogative questions (*β* = 0.23, *SE* = 0.05, *z* = 4.93*, p* < 0.001), between declarative and wh-questions (*β* = 0.17, *SE* = 0.04, *z* = 3.88*, p* = 0.001), between interrogative and non-clausal questions (*β* = − 0.34, *SE* = 0.11, *z* = − 3.03*, p* = 0.021), between interrogative and tag-questions (*β* = − 0.24, *SE* = 0.04, *z* = − 5.78*, p* < 0.001), and between tag- and wh-questions (*β* = 0.18, *SE* = 0.04, *z* = 4.75*, p* < 0.001).

## Discussion

In the present study, we investigated the contribution of eyebrow movements with natural movement intensities to the rapid identification of questions. Results showed higher accuracies for eyebrow frowns compared to the absence of eyebrow movements, and faster response times for eyebrow frowns and eyebrow raises compared to the absence of eyebrow movements. There was no effect of eyebrow raises for accuracy. No additional effect of specific eyebrow movement intensity was observed for accuracy nor for response time. Although there was only anecdotal evidence in support of a null effect of specific eyebrow movement intensity for accuracy, there was strong evidence in support of a null effect of specific eyebrow movement intensity for response time.

The finding that questions with eyebrow frowns resulted in higher accuracies and faster response times compared to questions without eyebrow movements is in line with previous studies showing an association between these eyebrow movements and questions^10,12,13,15–20,22^, and past experimental studies showing that eyebrow frowns help to identify questions^24–27^. This study therefore replicates previous findings showing that eyebrow movements facilitate question identification. Moreover, it extends previous studies by showing a facilitative effect of eyebrow frowns with timings and variable movement intensities based on spontaneous multimodal behaviour.

The faster response times observed for questions with eyebrow raises compared to questions without eyebrow movements show that when movement intensities are more representative of visual behaviour occurring in a multimodal corpus, eyebrow raises do in fact contribute to the rapid identification of questions, in line with previous studies showing an association of these eyebrow movements with questions^10,12–18,21,22^ and past experimental studies^14,26,28^. In Nota et al.^24^, no facilitation effect was observed for eyebrow raises. Thus, it may be that the natural variability of eyebrow movement intensity made eyebrow raises a clearer signal for questions, by being a closer match to the original visual behaviour in the corpus. The lack of a facilitative effect of eyebrow raises in Nota et al.^24^ was suggested to be caused by the common occurrence of eyebrow raises in other contexts (e.g., prosodic emphasis^31^), and by the weaker link of eyebrow raises with information requests compared to eyebrow frowns^13^. This argument may still apply, as shown by the lack of an effect of eyebrow raises for accuracy. Thus, eyebrow raises do seem to play a role when we consider natural movement intensities, but they may still be weaker signals for information requests than eyebrow frowns.

The lack of a modulating effect of specific eyebrow movement intensity for accuracy and response time suggests that the specific movement intensity may not matter for the recognition of questions, as long as the intensity is representative of actual multimodal behaviour. Thus, an intensity on a maximum setting may not be a clearer signal than an intensity on a lower setting (e.g., intensity level 5 versus 1), in line with the absence of a visual facilitation effect when the signals were enhanced^28^. This lack of a modulating effect of intensity was especially clear for response time, as shown by the strong evidence in support of a null effect of intensity. However, there is less conclusive evidence for a null effect of specific eyebrow movement intensity for accuracy.

As expected, there was an effect of question type and utterance duration for accuracy, and an effect of difference in onsets, eyebrow movement duration, question type, and utterance duration for response time, in line with Nota et al.^24^. This shows that there are linguistic ways to mark questions in the spoken modality, and that longer question durations make it easier to identify the utterance but slow down the response time (measured from the beginning of the video until the button-press). Participants responded faster to eyebrow movements occurring earlier, and faster to longer eyebrow movements, further demonstrating that long facial signals that foreshadow the verbal utterance are especially helpful in signalling social actions.

A limitation of the current study is that prosodic features were kept intact and were not further manipulated between conditions. Therefore, an interesting follow up would be to investigate the interaction between prosodic features and visual signals on fast question recognition. Moreover, all of the questions originally occurred with eyebrow movements in the corpus. For our experimental manipulation, these eyebrow movements were recreated based on the corpus in one condition, while they were left out in the other condition. This approach allowed us to preserve the natural (i.e., matching the speech) intensity and timing of the facial signals. Investigating whether adding eyebrow movements to questions that are not naturally associated with these facial signals would also result in facilitation of question identification would show whether eyebrow movements can cue questions more generally. While past studies^32^ have replicated common psycholinguistic effects from human–human interaction with avatars making it likely that the present findings, too, are generalisable beyond perceiving avatars asking questions, future research is required to investigate this systematically. Furthermore, investigating the effect of different social factors (such as individual differences between participants) on task performance could give more insights into individual variability in visual signal perception. Lastly, future studies looking into the temporal processing of facial signals and questions using neuroimaging measures could shed more light on the specific time-course of multimodal social action recognition.

To conclude, we found that eyebrow movements with natural intensities facilitate question identification. This finding demonstrates that facial signals, even while varying in their actual intensity or visual prominence, critically contribute to the communication of fundamental social actions like questions, and play an important role in multimodal human communication. Beyond providing new insights into how the face contributes to the dynamics of face-to-face conversation, this study is especially informative for the development of virtual characters such as avatars or social robots, and research on motion tracking.

## Methods

### Participants

We recruited 97 native speakers of Dutch between 18 and 45 years old through the subject database of the Radboud University in Nijmegen. Participants had no motoric, hearing, or language problems and normal or corrected-to-normal vision. A number of participants (*n* = 14) were manually excluded because they did not meet the specific requirements to participate in the current study. One additional participant was excluded to have a balanced set of individuals. This resulted in a final sample of 82 participants (mean age: 23 ± 5 years, 61 females, 19 males, 2 non-binary). Informed written consent was obtained prior to the study and a compensation of 7.50 euros was given at the end of the experiment. The study was approved by the Ethics Committee of the Social Sciences department of the Radboud University Nijmegen (ethic approval code ECSW 2018–135). Due to the global pandemic, non-invasive research was approved to be performed online. All methods were performed in accordance with the relevant guidelines and regulations.

To characterize our participant sample, we have provided an overview of participants’ Empathy Quotient (EQ) scores^33,34^, Actions and Feelings Questionnaire (AFQ) scores^35,36^, avatar evaluations designed to assess the participants’ perception of the avatars’ humanness, ease of understanding, and likeability^12,19,32,37^ in the Appendix (Supplementary Table S1).

### Stimuli

#### Corpus

**Figure 3:**
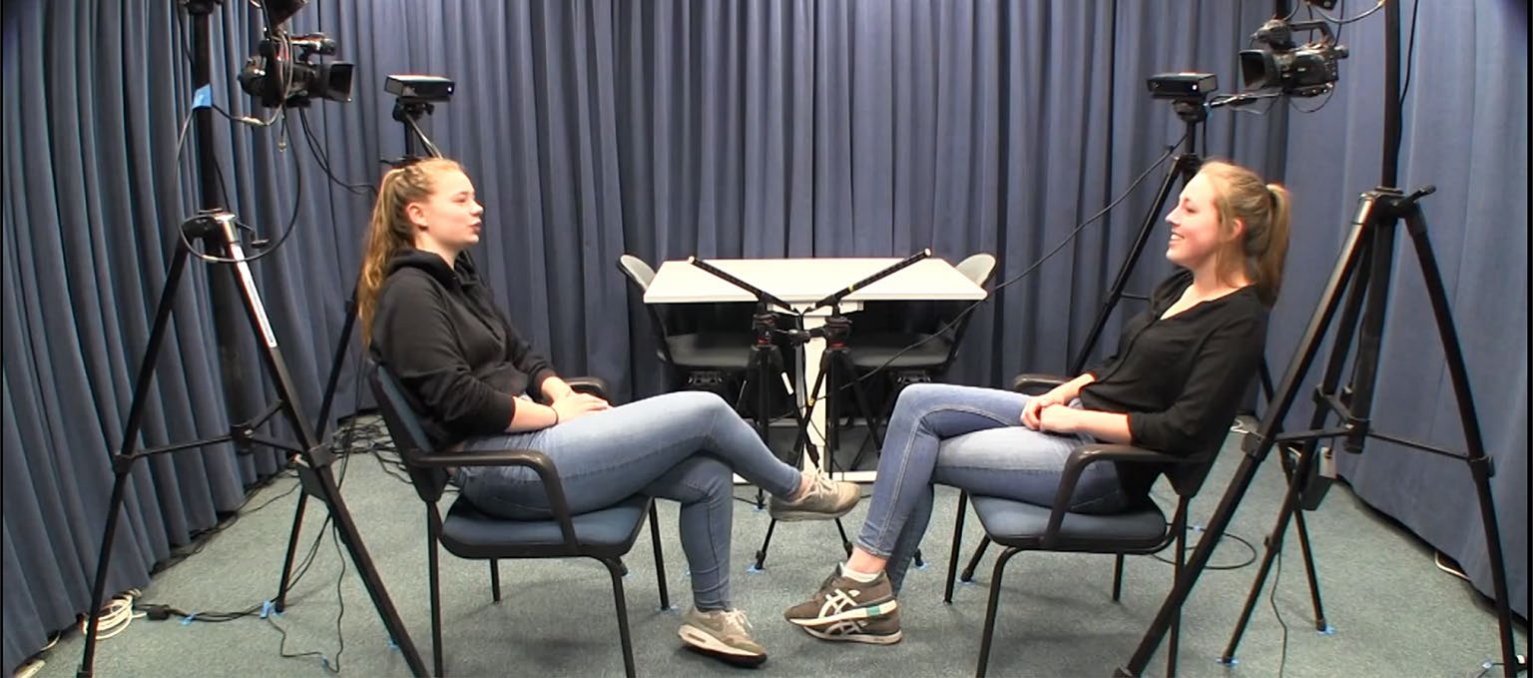
Overview set up from one dyad.

The same avatar stimuli were used as in Nota et al.^24^ (to the exceptions of the variable movement intensities), which were based on audio and video recordings of Dutch acquaintances holding a conversation for one hour in a face-to-face sitting arrangement (CoAct corpus, ERC project #773079 led by JH). Note that all CoAct corpus participants who are depicted in the figures of this manuscript provided informed written consent for the publication of their images in an online open-access publication. See Fig. 3 for an example of a still frame from one dyad.

Questions, responses, social actions of questions (e.g., information requests, which ask for new information of factual or specific nature), question types (polar or content, as well as types of polar questions), facial signals (like eyebrow frowns and raises), and eyebrow movement intensities were manually transcribed in ELAN^38^ (5.5) by Dutch speakers (see also ^12,13,24,39^ for more details on corpus conventions). The transcription of questions and responses largely followed the coding scheme of Stivers and Enfield^40^, with additional rules to account for the complexity of the corpus data. Context of the conversational exchange, communicative intention, and interactional response were considered. For questions and responses, interrater reliability between coders was assessed on 12% of the data with raw agreement and a modified Cohen’s kappa^41,42^ using EasyDiag^43^ with a standard overlap criterion of 60%. This resulted in a raw agreement of 75% and *k* = 0.74 for questions, 73% and *k* = 0.73 for responses, all indicating substantial agreement. Precise beginnings and endings of questions and responses were segmented using Praat^44^ (5.1) based on the criteria of the Eye-tracking in Multimodal Interaction Corpus^45,46^. A subset of 2082 questions were coded for their question type. Interrater reliability was measured on 686 additionally coded questions following the same procedure as for questions and responses. For question type, this resulted in a raw agreement of 98% and *k* = 0.97, indicating almost perfect agreement. Facial signals were annotated when they involved movements that carried some form of communicative meaning related to the questions and responses. It should be noted that transcribing corpus data manually is an extremely laborious process. Transcribing all facial signals (N = 34,413) frame-by-frame took approximately 1000 h. Therefore, for facial signals, interrater reliability was calculated by randomly selecting one question and one response from in one of the three recording parts for each participant in all dyads (*n* = 136, roughly equivalent to 1% of the question and response data) resulting in the additional annotation of 223 facial signals for reliability, and allowing a pairwise comparison between coders. This pairwise approach was chosen to account for the unequal amount of data that was transcribed between coders. The paired comparisons of the facial signals showed an average raw agreement of 76%, and an average *k* = 0.96, indicating almost perfect agreement.

For the current experiment, eyebrow movement intensities of the corpus recordings corresponding to the target stimuli used by Nota et al.^24^ were annotated on a 1–5 scale, one being barely any movement and five being an extremely high movement intensity. One of the authors (VJ) annotated all of the data constituting the basis for the stimuli (*n* = 80), and trained the second coder (AT) on 10%. AT was blind to the study hypotheses, and proceeded to code the remaining 90% independently. Interrater reliability was calculated between coders and showed a raw agreement of 81% and *k* = 0.76, indicating substantial agreement. Any disagreements between the two coders were resolved in the final version of the intensity annotations by discussion.

Eighty information request questions were selected from the corpus. Forty questions had eyebrow frowns (20 polar, 20 content), and 40 questions (20 polar, 20 content) had eyebrow raises. While there were cases in the corpus that had eyebrow frown-raise combinations, we only utilized utterances that either had an eyebrow frown or an eyebrow raise. Questions without eyebrow movements were created by stripping away the original eyebrow movements from the questions with eyebrow frowns and raises. Thus, the original eyebrow movements were recreated based on the corpus for one condition and left out in the other condition. To distract from the main experimental aim, we selected 80 statements as fillers. Twenty statements had eyebrow frowns, and 20 statements had eyebrow raises. Forty statements did not have eyebrow movements. These statements were selected from the same speakers as the questions were selected from, to have an equivalent number of questions and statements from each speaker. Moreover, the statements with an eyebrow movement (frown or raise) had the same type of eyebrow movement as the questions from the same speakers (e.g., question with frown from speaker 1 resulting in statement with frown from speaker 1 as well). To vary the visual signals, there were another 20 additional fillers (10 questions and 10 statements) accompanied by facial signals of the eyes (5 eye widenings, 5 squints). Most facial signals were found to occur before or at the start of the verbal utterance in the corpus^12^. Therefore, we only included questions and statements with eyebrow movements, eye widenings, and squints that occurred before or at the start of the verbal utterance (the mean difference between all facial signal onsets and verbal utterance onsets was 536 ms). Facial signal movement onsets varied within this time window, and there were different facial signal durations. This led to the inclusion of recordings of 53 speakers from the corpus (mean age: 22 ± 7 years, 41 females, 12 males) for the avatar animations.

#### Avatars

**Figure 4:**
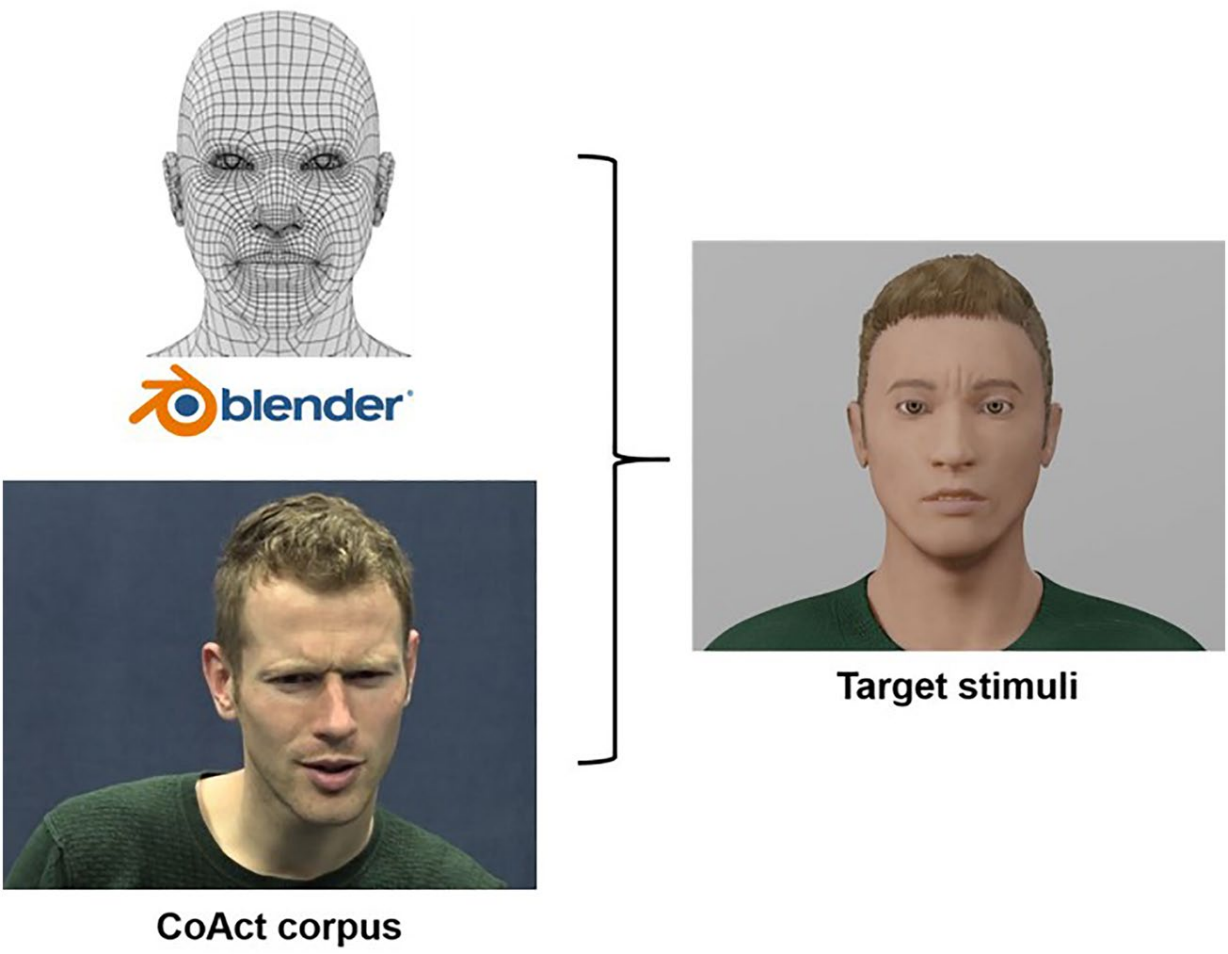
Simplified overview of avatar creation.

Avatars were created for the corresponding videos of each stimulus from the corpus. The avatars were created in Blender^47^ (2.83.12) using the MB Lab^48^ plug-in (1.7.6). The appearance of the avatar was matched to the original corpus speaker in terms of skin, hair, and clothing. Face detection was based on action units, gaze, and head pose of the corpus videos retrieved by OpenFace^49^ (2.2.0). This was imported in Blender with FACSvatar^50^ (version 0.3.4-alpha) using the FACSvatar-Blender^51^ plug-in (0.4.0). See Fig. 4 for a simplified overview of the avatar creation.

In addition to the facial signals in the stimuli, direct gaze and blinks were added manually. With the exception of gaze, the timing of all facial signals was extracted from the facial signal transcriptions of the corpus, and had a gradual fade in and fade out that was largely based on the original fade lengths. Facial signal fades were coded in ELAN^38^ (5.5) from the first evidence of movement until the movement peak, or from the movement peak until the last evidence of movement. Fades under two frames were made more gradual by changing them to 80 ms.

**Figure 5:**
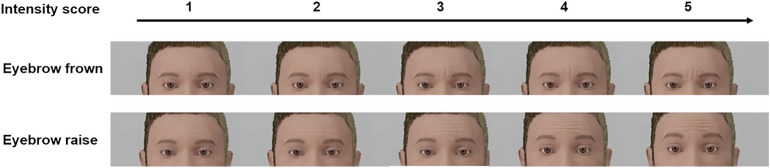
Example of the eyebrow movement intensity scores on a male avatar. In Nota et al.^24^, movement intensities were always of intensity score 5.

For the target stimuli, the intensities of eyebrow movements (frowns and raises) were matched to the natural intensities they had in the corpus by splitting the standard intensities generated by FACSvatar in five equal proportions (1 corresponding to the minimum value, 5 the maximum value, see Fig. 5). For the fillers (statements with eyebrow movements, and questions and statements with eye widenings or squints), a random intensity between 1–5 was used. The intensities were further adjusted to look proportionate to (and as similar between) the female and male avatar versions (see the Open Science Framework project website https://osf.io/e5gdm/ for the specific intensities). The movement intensities of gaze and blinks had fixed standard intensities generated via FACSvatar.

To avoid an abrupt start, we used ffmpeg^52^ (4.3.1) to add a still frame of a neutral face (without any visual signal) lasting 400 ms at the beginning of each video. Videos started with 400 ms of a neutral face, before the utterance onset. All visual signals started before or at the onset of the verbal utterance. For questions without eyebrow movement, videos continued at the original eyebrow movement onset, to keep durations consistent across conditions. For statements without any eyebrow movement, videos continued at the eyebrow movement onset from a randomly paired statement with an eyebrow movement, since there was no original eyebrow movement onset. The verbal utterance started at the original timing.

The avatars’ lip movements were synchronized with the speech files using a custom script in Python^53^ (3.7) with phoneme annotations, and manually edited. The precise beginnings and endings of the phonemes were segmented in Praat^44^ (5.1) using WebMAUS Basic^54^ (3.3) and manual transcription. These transcriptions were imported in ELAN^38^ (5.5), and exported as text files. Potential background noise was avoided by muting the sound from the beginning of the video until the start of the verbal utterance using ffmpeg^52^ (4.3.1). The overall sound intensity was normalized to 60 dB sound pressure level and converted to mono in Praat^44^ (6.1.40), to prevent loudness differences.

### Design

Stimuli were pseudo-randomized across two lists. Questions were presented in two conditions: a) with eyebrow movement (frown, raise), b) without eyebrow movement (frown or raise removed). Each participant saw each question only in one condition. Statements were always the same set in the two lists, meaning all participants saw each statement with the same eyebrow movement (or lack thereof). The two lists had a total of 180 trials. The experiment consisted of five blocks per list (with 16 questions, 16 statements, 4 fillers per block, and with a balanced number of utterances with and without visual signal per block), allowing participants to rest between blocks. The order of blocks was randomized, as well as the items within each block.

### Procedure

The experiment was conducted online using Gorilla^55^ (www.gorilla.sc). Participants were required to have Google Chrome and Windows to control for visual delay across different platforms^56^, and wired earphones or headphones, to avoid potential sound delays via Bluetooth. They were asked to sit in a quiet room behind a desk or table, and switch off electronics and notifications. Participants began with a general demographics and language background questionnaire. Then, a sound was played using an adapted version of the web-based headphone screening test^57^ to allow them to adjust their volume, and to make sure that autoplay was enabled. The task opened in full-screen, so that the videos (1280 × 720) scaled to the maximum monitor space.

Participants were instructed to indicate whether the utterance in the videos was a question (*vraag*) or a statement (*stelling*) as quickly and accurately as possible by using response keys “X” and “M”. They first performed 16 practice trials (8 questions and 8 statements, each with 1 eyebrow frown, 1 eyebrow raise, 2 without eyebrow movements, 2 squints, and 2 eye widenings). This was followed by the 180 experimental trials. Each trial started with a fixation cross in the middle of the screen for 500 ms, after which the video was played. As soon as there was a button-press, a blank screen was presented for 1000 ms, and then the next trial began. If there was no button-press, the video played in its entirety. In that case, the last video frame was shown at the end of the video until there was a button-press before moving to the blank screen. There were four self-paced breaks between blocks. Before being debriefed, participants filled in three psycho-social questionnaires^12,19,32–37^ and questions assessing awareness of the experimental aim. The entire experiment lasted approximately 45 min.

### Analysis

Participants’ accuracies and responses times (RTs) were first checked for outliers, which we considered to be RTs more than 2.5 *SD* from the mean participant RT. This resulted in the removal of 156 trials (2% of total trials). Inaccurate responses were excluded from the RT dataset (*n* = 1062, or 16% of RT dataset).

To see whether we could find an effect of natural eyebrow movement intensities, we first fitted generalised linear mixed-effect models (GLMMs) in *R*^58^ (4.1.2) with *RStudio*^59^ (2021.09.2–382) with the *glmer* function in the *lme4*^60^ package (1.1–28)*.* GLMMs allow the inclusion of additional predictors, and provide a solution for fitting the distributions by satisfying normality assumptions without the need for transformation^61^. The same fixed and random parameters were used in our models for each of the two dependent variables (accuracy, RT) as in Nota et al.^24^. These were selected on the basis of our research questions and experimental design, following the recommendations of Meteyard and Davies^62^. The fixed effects were eyebrow movement (with frown, with raise, without eyebrow movement), the difference between eyebrow movement onset and utterance onset (scaled), eyebrow movement duration (scaled), question type (declarative, interrogative, non-clausal, tag-, wh-), and utterance duration (scaled). The reference levels were absence of eyebrow movement and wh-question type. We included random intercepts by participant and item, and did not add random slopes since this led to convergence issues in the power analysis based on pilot data, and would unnecessarily add complexity to the models^62^. We then ran log-likelihood ratio tests (ANOVA function) to test for the presence of main effects for the fixed parameters. A post-hoc analysis was performed for eyebrow movement and question type, using the Tukey method with *emmeans*^63^ (1.7.2). In all models, we applied Helmert contrasts for the eyebrow movement variable, so that the first contrast compared absence of eyebrow movement with presence of eyebrow frown, and the second contrast compared absence of eyebrow movement with presence of eyebrow raise.

To see if there was an effect of eyebrow movement intensity, we ran new GLMMs with the same fixed and random parameters for each of our two dependent variables as our previous models, including eyebrow movement intensity (1–5) as a fixed parameter. We then ran log-likelihood ratio tests (ANOVA function) to test for the presence of a main effect of eyebrow movement intensity. To find out whether there was evidence for the null hypothesis, we additionally fitted Bayesian linear mixed models in *R*^58^ (4.1.2) with the *brm* function in the *brms*^64^ package (2.18.0) with the same fixed and random parameters for each of our two dependent variables as the GLMMs including eyebrow movement intensity. We computed Bayes factors to estimate whether the data favoured a model without eyebrow movement intensity over a model with eyebrow movement intensity.

An exploratory analysis assessing the relation between accuracy and RT can be found in the Appendix (Supplementary Fig S2). The analysis script and additional session information can be found on the Open Science Framework project website https://osf.io/e5gdm/.

### Supplementary Information

Supplementary Information.

## Data Availability

The analysis scripts with session information, depersonalized data, and results are available on the Open Science Framework project website https://osf.io/e5gdm/.
