# Supplementary material for: Facilitating question identification through natural intensity eyebrow movements in virtual avatars
Source: Sci Rep. 2023 Dec 2;13:21295. doi: 10.1038/s41598-023-48586-4 (PMC10693605; doi:10.1038/s41598-023-48586-4)

**Supplementary Table S1.** Participant characteristics.

| Characteristic | Mean (*SD*) | Minimum and maximum possible score |
| --- | --- | --- |
| EQ | 43 (10) | 0-80 |
| AFQ | 34 (6) | 0-54 |
| Avatar evaluation | 7 (3) | 0-15 |
| *1) Humanness* | 2.28 (1.25) | 0-5 |
| *2) Ease of understanding* | 2.39 (1.17) | 0-5 |
| *3) Likeability* | 2.17 (1.35) | 0-5 |

To assess whether there was a relation between accuracy and response time, we ran additional GLMMS with the same fixed and random parameters as our original accuracy model, and added response time (scaled) as a fixed effect. We then ran a log-likelihood ratio test (ANOVA function) to test for an effect of response time. We found that there was a significant effect of response time for accuracy (*χ*^2^(1) = 19.32, *p* < .001), showing that longer response times resulted in lower accuracy scores. Thus, there does not seem to be a speed-accuracy trade-off for eyebrow frowns nor for eyebrow raises, since participants were not faster and less accurate or slower and more accurate at detecting questions with these eyebrow movements.

**Supplementary Fig S2.** Speed-Accuracy trade-off. The x-axis shows the mean response time for each signal (no eyebrow movement, eyebrow frown, eyebrow raise) per participant, and the y-axis the mean accuracy for each signal per participant.


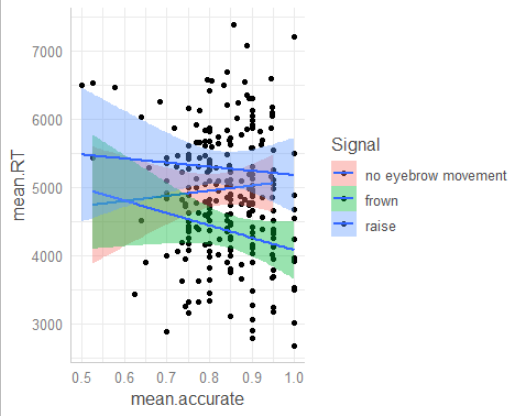

Supplement: Supplementary file 1 — Supplementary Information. [file 41598_2023_48586_MOESM1_ESM.docx]
